# Improving the efficacy of phenolic extract from *Pimpinella affinis* in edible oils through nanoencapsulation: Utilizing chitosan and *Salvia macrosiphon* gum as coating agents

**DOI:** 10.1002/fsn3.4179

**Published:** 2024-04-29

**Authors:** Habib Abbasi, Javad Tavakoli, Fahimeh Zare, Mohsen Salmanpour

**Affiliations:** ^1^ Department of Chemical Engineering Jundi‐Shapur University of Technology Dezful Iran; ^2^ Department of Nutrition Sciences, Ewaz School of Health Larestan University of Medical Sciences Larestan Iran; ^3^ Department of Food Science and Technology, Faculty of Agriculture Jahrom University Jahrom Iran; ^4^ Cellular and Molecular Biology Research Center Larestan University of Medical Sciences Larestan Iran

**Keywords:** chitosan, extract, nanoencapsulation, *Pimpinella affinis*, *Salvia macrosiphon* gum

## Abstract

In the present study, a phenolic extract derived from the *Pimpinella affinis* plant underwent nanoencapsulation. The nanoencapsulation process employed chitosan, *Salvia macrosiphon* gum (SMG), and a chitosan–SMG complex (1:1) (CCS) as coating agents. The evaluation of nanoemulsions encompassed measurements of particle size, polydispersity index (PDI), ζ‐potential, encapsulation efficiency, and intensity distribution parameters. The overall results of these assessments indicated that the nanoemulsion coated with CCS exhibited the most favorable characteristics when compared to other treatments. Subsequently, this specific nanoencapsulated sample was utilized to enhance the oxidative stability of canola oil at concentrations of 100, 200, and 300 ppm (parts per million). Oxidative stability tests, assessed through the total oxidation value (TOTOX) index, highlighted the superior performance of the nanoencapsulated extract, particularly at a concentration of 300 ppm. This enhancement can be attributed to the increased release of phenolic compounds from the CCS coating into the canola oil. The findings illustrate that the nanoencapsulation process can significantly enhance the efficacy of *P. affinis* extract in improving the oxidative stability of canola oil.

## Graphical abstract

The use of antioxidants in foods to prevent the activity of free radicals is essential and increases the useful life of edible oils, but an important problem faced in the effective use of antioxidants is that these compounds are added to oils and food at once. In the early stages of edible oils' storage, in order to prevent the oxidation reaction, a small amount of these antioxidants is needed. The best way to improve the activity of antioxidants is to create conditions that gradually add them to foods. Today, the use of encapsulation technique is expanding to improve the functional properties of bioactive compounds including antimicrobial and antioxidant activity. Results of the present study indicated that the use of material coatings to nanoencapsulation of antioxidants improved their antioxidant activity.

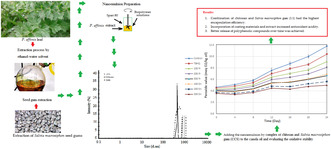

## 1 INTRODUCTION

The principal factor leading to spoilage in edible oils and fats is the occurrence of oxidative reactions, which not only impart an unpleasant odor and taste but also yield undesirable compounds like hydroperoxides and carbonyls (Dunford, 2015; Mahjoob et al., 2018). Moreover, these oxidation processes can generate free radicals, which can adversely affect biological cells and contribute to inflammatory issues in humans (Amiri et al., 2021). To address these concerns, the addition of antioxidant compounds has become a common practice in preserving the quality of edible oils (Hosseinialhashemi et al., 2021).

Contemporary research endeavors have increasingly focused on the adoption of natural antioxidants as alternatives to chemical counterparts (Chong et al., 2015; Es et al., 2017). Among these natural antioxidants, phenolic extracts derived from plants hold particular significance (Generalić Mekinić et al., 2014; Kozłowska & Gruczyńska, 2018; Oliveira et al., 2018). However, their practical use in food faces various challenges, including low solubility and vulnerability to environmental factors (Mohamadi et al., 2023; Tavakoli et al., 2023).

In response to these challenges, researchers have proposed the application of the nanoencapsulation process for phenolic compounds. Nanoencapsulation involves the encapsulation of plant extracts, rendering them more amenable for integration into various food ingredients (Mohammadi et al., 2016; Mohammadi et al., 2015; Munin & Edwards‐Lévy, 2011). This approach offers a promising solution to enhance the stability and functionality of phenolic extracts in food applications.

Recently, there has been a growing trend toward using natural polymers for the nanoencapsulation of antioxidant compounds, a strategy that enhances their activity through controlled release. Among the various natural compounds utilized for this purpose, chitosan and gums have gained prominence as effective coatings in the nanoencapsulation process (Jamshidi et al., 2020; Tavakoli et al., 2021). *Salvia macrosiphon* gum (SMG) is one of the available gums in Iran that show promise for nanocoating applications (Tavakoli et al., 2021).

*Salvia macrosiphon*, a member of the mint family, yields gum containing primarily mannose and galactose, with smaller quantities of glucose, arabinose, and rhamnose (Bostan et al., 2010). A study by Tavakoli et al. (2023) employed chitosan and *Lepidium perfoliatum* seed gum for the nanoencapsulation of phenolic extracts from *Mentha aquatica*. The results demonstrated that extracts nanoencapsulated with *L. perfoliatum* seed gum at a concentration of 200 ppm exhibited the highest oxidative stability in canola oil.

Estakhr et al. (2020) and Delfanian et al. (2018) have also reported favorable outcomes from the nanoencapsulation process. Estakhr et al. (2020) focused on the phenolic extract of *Ferula persica*, while Delfanian et al. (2018) investigated the nanoencapsulation of *Pistacia atlantica* hull oil. In both cases, the process significantly improved the antioxidant activity of the respective compounds.

Anarijeh, scientifically known as *Pimpinella affinis*, is a biennial plant native to Iran, belonging to the *Apiaceae* family and *Pimpinella* genus. Its geographical distribution encompasses Anatolia, Iran, Turkmenistan, Afghanistan, Caucasus, and Iraq. In Iran, this plant is found in various regions, including the northern, northwestern, central, eastern, and northeastern areas (Mozaffarian, 2012). Species within the *Pimpinella* genus have diverse applications in different regions of Europe, Asia, and Iran. For instance, *Pimpinella anisum* is used as an expectorant, a milk enhancer, a disinfectant, an antiflatulent agent, and as an additive in the beverage industry. In Germany, *Pimpinella saxifraga* is used in expectorant medications, and in Turkey, it is known as a soothing and antiflatulent remedy. *Pimpinella major* is marketed in Australia as an antibacterial medicine (Sarrafi et al., 2018).

The *P. affinis* plant contains various phenolic compounds with antioxidant properties. Notably, it is rich in compounds such as flavonoids and sulfur‐containing substances like diallyl sulfide and trisulfide, as well as allylcysteine, all of which have been recognized for their significant biological effects (Özbek et al., 2016; Tharun & Kumar‐Pindi, 2013). Consequently, the phenolic extract from *P. affinis* holds promise as a natural antioxidant for use in edible oils.

It is worth noting that no prior research has explored the nanoencapsulation of the phenolic extract from this plant. Therefore, the objective of this study is to investigate the impact of the nanoencapsulation process on the antioxidant properties of the phenolic extract obtained from *P. affinis*, using chitosan and *S. macrosiphon* seed gum as coating agents.

## 2 MATERIALS AND METHODS

### 2.1 Materials

*Pimpinella affinis*, sourced from Vasteriosh company in Sari City (Mazandaran, Iran), served as our primary plant material. *Salvia macrosiphon* gum was prepared using 6 kg of seeds procured from Tabib Daru Company in Shiraz. Canola oil, devoid of antioxidants, was obtained through collaboration with Ghoncheh Sari Oil Company and utilized as the base oil in this research. Essential solvents and chemicals were sourced from Merck and Sigma‐Aldrich companies.

### 2.2 Extraction process

To prepare the phenolic extract, 200 g of powdered *P. affinis* was blended with a 50:50 ethanol/water solvent mixture (500 mL). Subsequently, the samples were subjected to ultrasonic treatment in a bath operating at 35 kHz for a duration of 27.5 min at a temperature of 45°C (Estakhr et al., 2020; Roshanpour et al., 2021).

### 2.3 Extraction of *Salvia macrosiphon* seed gums

The *S. macrosiphon* seed gum was extracted from whole seeds using distilled water (water‐to‐seed ratio of 55:1) at pH 5.5 according to Bostan et al. (2010). Extraction was carried out in three stages; in the first stage, the seeds were mixed with water and enough time (20 min) was given that complete water absorption occurred. A soaking time of 20 min was selected based on the yield of preliminary trials. Separation of the gum from the swelled seeds was done by passing the seeds through a laboratory extractor. Crude gum was collected and residual seeds were immersed in the remaining water in two stages, according to the water‐to‐seed ratio proposed for each run and again this mixture was passed through the extractor. The collected crude gum from the different stages was mixed, filtered, and dried overnight in a forced convection oven.

### 2.4 Preparation of biopolymer solutions

Wall materials solution (0.5% w/v) was prepared by dissolving *S. macrosiphon* seed gum in the distilled water and chitosan in the acetic acid solution (1%) and stirring for 30 min at room temperature. The solutions were kept in the refrigerator for 24 h to complete the hydration and disengage the bubbles (Tavakoli et al., 2023).

### 2.5 Preparation of nanoemulsions

Nanoemulsions are prepared using the method described by Jamshidi et al. (2020).

### 2.6 Particle size measurement and ζ‐potential

Particle distribution, z‐average particle size, and polydispersity index (PDI) were determined using a Dynamic Light Scattering (DLS) instrument at 25°C, as previously described by Tavakoli et al. (2021) and Mohammadi et al. (2016). The measurement of ζ‐potential was carried out according to the method established by Mohammadi et al. (2015).

### 2.7 Freeze‐drying of nanoemulsions

The nanoemulsions, prepared as described in the previous step, were subjected to freeze‐drying. Initially, they were placed in a freezer at −50°C for one night and subsequently dried in a freeze‐dryer under controlled conditions with a pressure of 0.09 bar and a temperature of 0.01°C for a duration of 48 h. Finally, the resulting products were processed into a powdered form using a mortar (Delfanian et al., 2018).

### 2.8 Encapsulation efficiency and total phenolic content

The determination of encapsulation efficiency followed the procedure outlined by Robert et al. (2010). The quantification of the total phenolic content in various samples was performed using the method described by Delfanian et al. (2018).

The encapsulation efficiency was calculated using the following formula:

Encapsulation efficiency (%) = 100 − ((*P*_2_/*P*_1_) × 100).

*P*_2_: surface phenolic compounds.

*P*_1_: theoretical total polyphenols content.

### 2.9 Release kinetics

To assess the stability of the nanocoatings, the release of phenolic compounds from the nanoemulsions was monitored. A total of 12 g of samples were stored at 30°C for a period of 24 days. At the conclusion of each week, the quantity of surface phenolic compounds was determined, following the method outlined by Roshanpour et al. (2021).

The constants of velocity (k) and time of half‐life (t1/2) were determined using the methodology described by Najafi et al. (2011).

### 2.10 p‐Anisidine value, peroxide value, and TOTOX value

The p‐Anisidine value and peroxide value tests were conducted in accordance with the methodologies recommended by Roshanpour et al. (2021) and Tavakoli et al. (2021), respectively. The total oxidation (TOTOX) value, utilized for estimating the oxidative deterioration of lipids, was defined as the sum of both values (peroxide value and p‐Anisidine value) for a comprehensive assessment of oxidation and was calculated using the following formula (De Abreu et al., 2010):

TOTOX value = (2 × peroxide value) + p‐Anisidine value.

### 2.11 Statistical analysis

The experiments in this research were conducted with three replications, and the obtained results were subjected to analysis of variance (ANOVA) for statistical evaluation. Furthermore, graphical representations and regression analysis were generated using Excel and Slide Write software.

## 3 RESULT AND DISCUSSION

### 3.1 Evaluation of nanoemulsions

The z‐average size of nanoemulsions is a crucial parameter for assessment, with smaller sizes being more desirable. To achieve reduced particle size in nanoemulsions, a range of pressures are applied, and the pressure leading to the smallest particle size is determined. In this study, pressures ranging from 9000 to 12,000 psi (pounds per square inch) were employed for 3, 5, and 7 cycles (each cycle lasting 90 seconds), and the optimal conditions were observed at a pressure of 10,000 psi (see Table 1). Beyond this optimal pressure, an increase in pressure led to an increase in droplet size in the emulsions. This phenomenon is attributed to re‐coagulation, a phenomenon noted in other studies as well (Delfanian et al., 2018; Estakhr et al., 2020). Table 2 reveals that the smallest particle size in nanoemulsions was achieved when using chitosan and *S. macrosiphon* gum (SMG) as coatings (109.7 and 121.2 nm, respectively) with a 5‐cycle process. In contrast, for the sample coated with a 1:1 complex of chitosan and SMG (CCS), the smallest droplet size was observed in a 7‐cycle process, measuring 69.2 nm. Hence, based on this evaluation, nanoemulsions coated with CCS demonstrated the most favorable characteristics among all samples. Interestingly, in some prior studies, it has been reported that the combined use of chitosan and gums led to optimal conditions for nanoemulsion production (Dehghan et al., 2020; Estakhr et al., 2020; Roshanpour et al., 2021). However, this is not always true. For instance, Tavakoli et al. (2023), in their research involving the nanocoating of *M. aquatica* phenolic extract using chitosan and *L. perfoliatum* seed gum, reported that the smallest droplet size in emulsions was observed in the sample coated with *L. perfoliatum* seed gum. Differences in emulsifying properties (surface activity, plasticity properties, etc.) of coating materials can cause different results in the droplet size of emulsions.

**TABLE 1:** Particle size of water‐in‐oil‐in‐water (W/O/W) double emulsions stabilized by different wall materials at three‐time cycles and pressure of 7 pounds per square inch (psi).

Sample	Pressure	Time cycle		
		3	5	7
Chitosan	9000	149.2 ± 0.6	128.9 ± 0.4	165.9 ± 0.3
	9500	137.1 ± 0.9	121.2 ± 0.3	148.4 ± 0.2
	10,000	125.6 ± 1.2	109.7 ± 0.8	137.6 ± 0.9
	10,500	133.4 ± 0.5	123.4 ± 0.6	146.1 ± 0.3
	11,000	152.5 ± 0.6	142.8 ± 0.7	163.2 ± 0.8
	11,500	168 ± 1.1	155.3 ± 0.6	191.3 ± 0.6
	12,000	185.2 ± 0.4	175.3 ± 0.9	208 ± 0.5
CCS	9000	96.1 ± 0.6	99.2 ± 0.6	88.1 ± 1
	9500	88.4 ± 0.5	89.3 ± 0.4	77.6 ± 1
	10,000	75.8 ± 0.8	79.9 ± 0.9	69.2 ± 0.9
	10,500	89.1 ± 0.8	88.1 ± 0.4	81.2 ± 0.6
	11,000	93.3 ± 0.7	95.2 ± 0.6	93.2 ± 0.5
	11,500	100.1 ± 0.6	103.2 ± 0.7	100 ± 0.4
	12,000	105.6 ± 0.5	109.7 ± 0.6	104.2 ± 0.6
SMG	9000	183 ± 1	145.2 ± 0.3	175.4 ± 0.9
	9500	162.5 ± 0.6	133.1 V0.6	162.1 ± 0.8
	10,000	144.2 ± 1.7	121.15 ± 1.1	141.4 ± 1.5
	10,500	151.3 ± 0.5	127.3 ± 0.5	154.3 ± 1.1
	11,000	181.6 ± 0.9	146 ± 0.2	177.3 ± 0.7
	11,500	194.3 ± 0.8	161.1 ± 0.5	189.3 ± 0.2
	12,000	207 ± 1.2	177.3 ± 0.5	199.5 ± 0.3

Abbreviations: CCS, complex of chitosan, and SMG (1:1); SMG, *Salvia macrosiphon* gum.

**TABLE 2:** Particle size, polydispersity index (PDI), and zeta‐potential of water‐in‐oil‐in‐water (W/O/W) double emulsions stabilized by different wall materials at three‐time cycles.

Sample	Particle size (nm)			PDI			ζ‐potential
	3	5	7	3	5	7	(mV)
Chitosan	125.6 ± 1.2^b^	109.7 ± 0.8^b^	137.6 ± 0.9^b^	0.44 ± 0.004^a^	0.48 ± 0.005^a^	0.42 ± 0.005^a^	25.6 ± 0.2^a^
CCS	75.8 ± 0.8^c^	79.9 ± 0.9^c^	69.2 ± 0.9^c^	0.25 ± 0.006^c^	0.3 ± 0.006^c^	0.34 ± 0.004^b^	−8.9 ± 0.1^b^
SMG	144.2 ± 1.7^a^	121.15 ± 1.1^a^	141.4 ± 1.5^a^	0.37 ± 0.007^b^	0.36 ± 0.005^b^	0.43 ± 0.006^a^	−40.1 ± 0.2^c^

*Note*: Means ± SD (standard deviation) within a column with the same lowercase letters is not significantly different at *p* < .05.

Abbreviations: CCS, complex of chitosan and SMG (1:1); SMG, *Salvia macrosiphon* gum.

**FIGURE 1:**
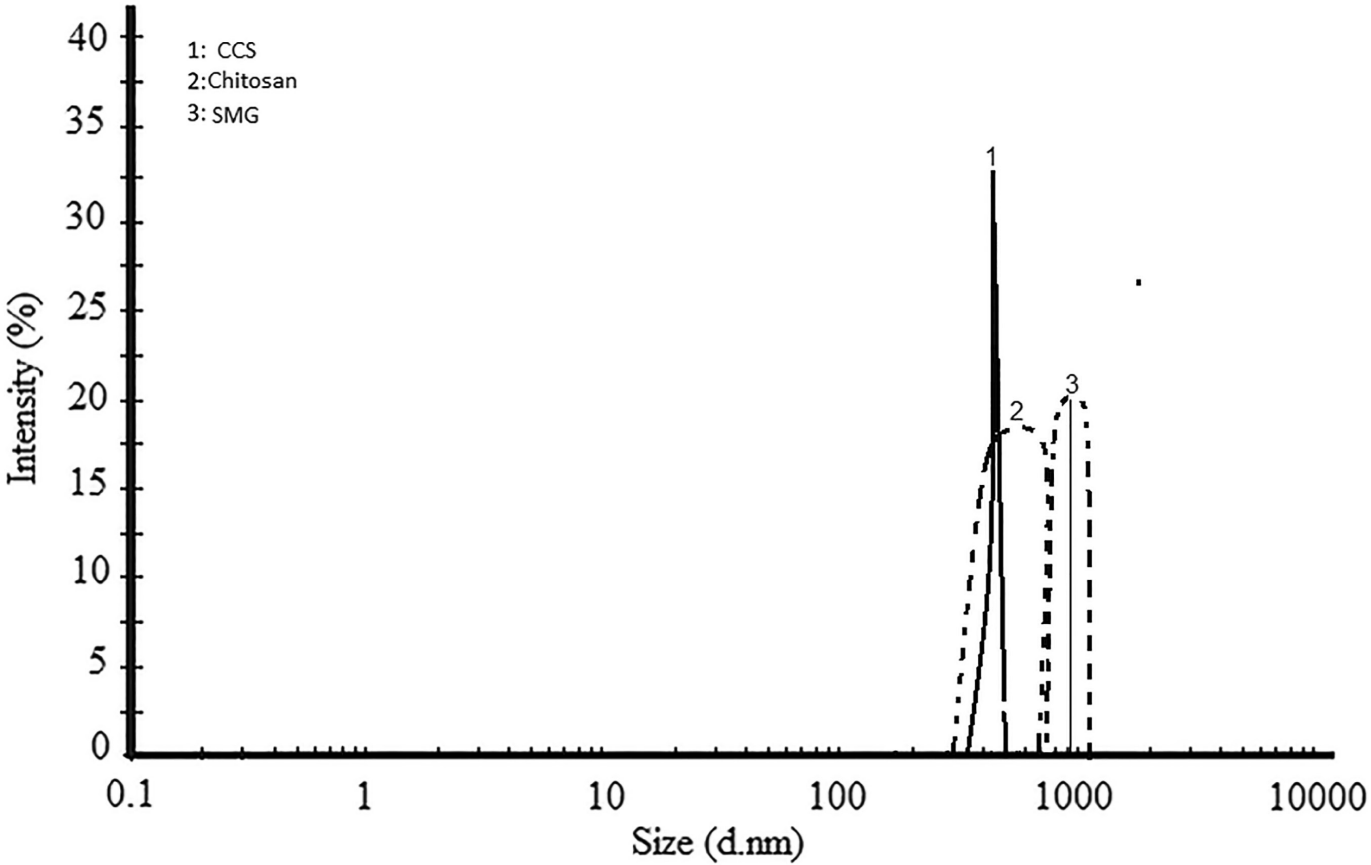
Particle size distribution of water‐in‐oil‐in‐water (W/O/W) emulsions of single‐layer chitosan and SMG and two‐layer CCS. CCS, complex of chitosan and SMG (1:1); SMG, *Salvia macrosiphon* gum.

Figure 1 shows the droplet size distribution of the emulsions prepared in this study based on the intensity parameter. The intensity distribution factor is considered more precise and significant than the z‐average size parameter. While the z‐average size is primarily a physical measurement, the intensity distribution factor is qualitative and holds greater importance. When examining the intensity distribution curve of the generated nanoemulsion, the presence of a peak suggests uniformity in droplet size distribution. Furthermore, the curve enables an understanding of the range of variations in the size of nanoemulsions. The smaller the width of the band, the smaller the range of changes in the droplet size of emulsions (Delfanian et al., 2018; Roshanpour et al., 2021). In this study, it was found that nanoemulsions coated with chitosan, CCS, and SMG had a peak at 570, 420, and 920 nm, respectively. Notably, the sample prepared with CCS exhibited the narrowest bandwidth, followed by the samples produced with SMG and chitosan, respectively. A significant aspect of this analysis is the correlation between the results of the intensity distribution curve and the actual droplet size of the emulsions in this research. This concordance validates the reliability of the intensity distribution curve as an indicator of droplet size uniformity and range within the nanoemulsions.

In two other studies, the evaluation of the intensity distribution curve led to the conclusion that the combined use (1:1) of chitosan and Qodume Shirazi gum and chitosan with locust bean gum for the microcoating process yielded the most favorable conditions (Estakhr et al., 2020; Roshanpour et al., 2021). Similarly, Tavakoli et al. (2021) reported that, in their research, the combined use of two gums, *Lepidium sativum* and *S. macrosiphon* seed gums (1:1), resulted in the best conditions based on the intensity distribution parameter. However, it iss essential to note that the combined use of coatings does not consistently guarantee the best outcomes in this evaluation. For instance, Tavakoli et al. (2023) reported that the emulsion coated with *L. perfoliatum* gum outperformed other samples in the intensity distribution test.

The polydispersity index (PDI) is indeed a crucial parameter for assessing emulsions, as it provides valuable information about the uniformity of particle size distribution. The range for PDI values typically falls between zero and 1. A PDI close to zero signifies a highly homogeneous dispersion of particle sizes, while a PDI greater than 0.5 suggests nonuniform conditions (Lutz et al., 2009). An examination of the results presented in Table 2 reveals that the PDI values for nanoemulsions under all conditions were less than 0.5. This indicates a uniform size distribution and underscores the success of the nanoparticle production process. Based on this test, it is apparent that the emulsions coated with CCS (ranging from 0.25 to 0.34) were the best sample. These values, being closer to zero, indicate a higher degree of uniformity when compared to the other two nanoemulsions across all time cycles. Following CCS, the emulsion coated with SMG (ranging from 0.36 to 0.43) exhibited favorable uniformity, with chitosan‐coated emulsions (ranging from 0.42 to 0.48) following as the next best option. This assessment underscores the superior performance of CCS‐coated emulsions in terms of particle size distribution uniformity. Tavakoli et al. (2023) reported that based on the PDI index, nanoemulsions coated with *L. perfoliatum* gum were the best among different treatments, with PDI values ranging from 0.36 to 0.46. Similarly, in separate studies, Estakhr et al. (2020) and Roshanpour et al. (2021) found that the combined use of chitosan with locust bean gum and chitosan with Qodume Shirazi gum, in a 1:1 ratio, resulted in optimal conditions based on the PDI test. Additionally, Tavakoli et al. (2021) reported PDI values for nanoemulsions coated with *Lepidium sativum* gum and *S. macrosiphon* gum, as well as the mixture of these two gums (in a 1:1 ratio). The PDI values ranged from 0.0996 to 0.1198, 0.1002 to 0.1123, and 0.072 to 0.091, respectively. These results highlight the benefits of using a combination of two gums, with the 1:1 mixture achieving the most favorable PDI values, indicating superior uniformity in particle size distribution.

In a colloidal system, the ζ‐potential represents difference between the immobile ion layer (Stern layer) and the mobile layer (diffusion layer) in the ionic atmosphere surrounding the charged. ζ‐potential is the highly informative indicator for determining the electrical state of the particle surface (Jones et al., 2010). In this study, the ζ‐potential values for nanoemulsion droplets coated with chitosan, CCS, and *S. macrosiphon* gum (SMG) were measured as 25.6, −8.9, and 40.1, respectively (see Table 2). As reported by Delfanian et al. (2018), emulsions are considered sufficiently stable over an extended period if their ζ‐potential falls within the range of +30 to −30. Accordingly, based on this factor, the emulsions coated with chitosan and CCS were found to exhibit greater stability than the emulsion coated with SMG.

The ζ‐potential values reported by Tavakoli et al. (2023) for emulsions coated with chitosan, *L. perfoliatum* gum, and the 1:1 complex of these two coatings (26.3, −35.3, and 18.1, respectively) are consistent with the results obtained in the present study. In another study, the ζ‐potential values for emulsion droplets coated with *Lepidium sativum* and *S. macrosiphon* seed gums were determined as −18.4 and − 18, respectively. Additionally, Estakhr et al. (2020) and Roshanpour et al. (2021) reported ζ‐potential values for emulsion droplets coated with the 1:1 complex of chitosan and locust bean gum and chitosan and Qodume Shirazi gum were −9.2 and 20.2, respectively. These findings further illustrate the impact of different coatings on the ζ‐potential of emulsions and their implications for stability.

The nanoencapsulation process plays a crucial role in enhancing the activity of antioxidant compounds, particularly phenolic extracts, by facilitating their controlled and gradual release from the selected coatings (Razali et al., 2012; Tavakoli et al., 2023).

Table 3 provides insight into the changes in encapsulation efficiency, which is based on the total polyphenols content, of extracts coated with different compounds during 24 days of storage at 30°C. It is important to note that the choice of coating material significantly influences the encapsulation efficiency. The initial encapsulation efficiency of the phenolic extract coated with chitosan, CCS, and SMG was measured at 81.1%, 87.6%, and 85.3%, respectively. These values represent the effectiveness of the coatings in retaining and releasing the phenolic compounds over time, thereby contributing to the improved activity of the antioxidants.

**TABLE 3:** Encapsulation efficiency, regression analysis, and half‐life values of encapsulated powders produced with different wall materials during 24 days of storage at 30°C.

Sample	Storage time (Day)						Parameters		
	4	8	12	16	20	24	K (Day^−1^)	t_1/2_ (Day)	*R* ^2^
Chitosan	81.1 ± 0.4^c^	78.3 ± 0.4^c^	75.1 ± 0.2^c^	70.0 ± 0.2^c^	67.3 ± 0.2^c^	63.0 ± 0.8^c^	0.0127	54.6	.9891
CCS	87.6 ± 0.6^a^	85.7 ± 0.6^a^	83.7 ± 0.3^a^	80.4 ± 0.9^a^	78.5 ± 0.5^a^	77.1 ± 0.6^a^	0.0098	70.7	.9696
SMG	85.3 ± 0.3^b^	81.3 ± 0.6^b^	76.6 ± 0.6^b^	73.8 ± 0.3^b^	72.2 ± 0.2^b^	70.1 ± 0.8^b^	0.0067	103.4	.9891

*Note*: Means ± SD (standard deviation) within a column with the same lowercase letters is not significantly different at *p* < .05.

Abbreviations: CCS, complex of chitosan and SMG (1:1); SMG, *Salvia macrosiphon* gum.

Tavakoli et al. (2023) reported initial encapsulation efficiency values for the phenolic extract of *M. aquatica* coated with chitosan and *L. perfoliatum* gum ranging between 76.1% and 85.9%. In separate studies, Roshanpour et al. (2021) and Estakhr et al. (2020) reported the highest initial nanoencapsulation efficiency for the phenolic extract of *Mentha piperita* coated with a chitosan–Qodume Shirazi complex (1:1) and the phenolic extract of *F. persica* coated with the combination of chitosan and locust bean gum (1:1). These values were notably high, at 93.3% and 90.8%, respectively, in comparison to other coatings.

The evaluation of changes in encapsulation efficiency reveals important insights into the stability and performance of nanoencapsulated extracts. In this research, it was observed that the extract coated with CCS exhibited the lowest reduction in encapsulation efficiency (12%), followed by the extract coated with SMG (17.8%) and chitosan (22.3%). This finding holds significance because it aligns with the results of other evaluations of nanoencapsulated extracts. Previous research has demonstrated that a reduction in droplet size in emulsions leads to increased nanoencapsulation efficiency for powders. In the current study, the emulsions coated with CCS had the smallest droplet size, which contributed to the highest encapsulation efficiency.

Tavakoli et al. (2023) reported in a study that the encapsulation efficiency of the phenolic extract coated with chitosan and *L. perfoliatum* gum ranged from 21.7% to 25.3%, which was higher than the values found in the present study. Additionally, Roshanpour et al. (2021) and Estakhr et al. (2020) reported in two different studies that the reduction in nanoencapsulation efficiency of *M. piperit*a extract coated with chitosan and *Alyssum homolocarpum*, as well as *F. persic*a extract coated with chitosan and locust bean gum after 24 days of storage at 30 degrees Celsius, ranged from 19.9% to 24.3% and 16.5% to 24.3%, respectively. These findings highlight the complex and multifaceted factors affecting encapsulation efficiency and the stability of nanoencapsulated extracts.

**FIGURE 2:**
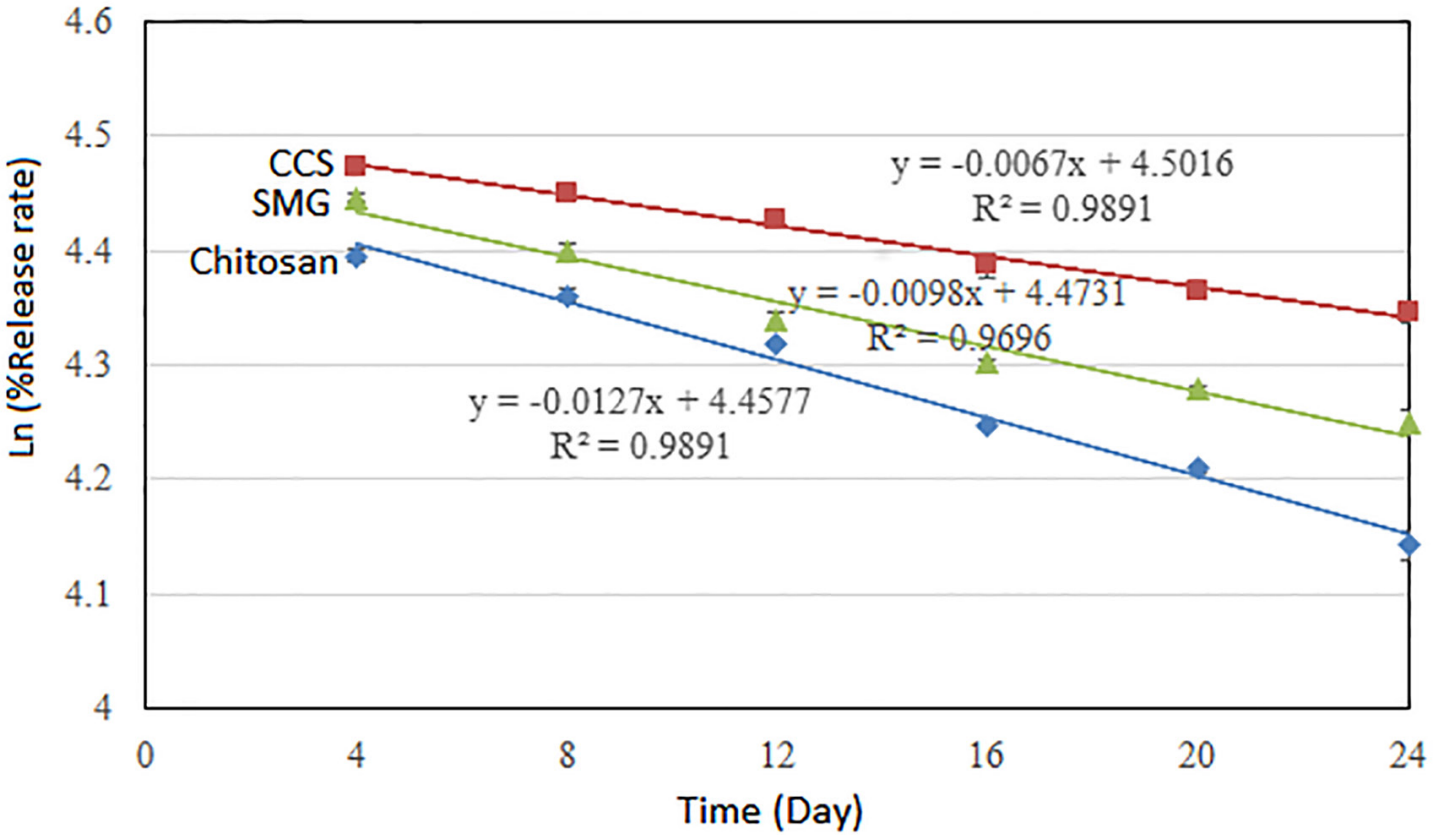
Release rate and regression equations of phenolic compounds of nanocapsules coated with SMG, chitosan, and CCL. CCS, complex of chitosan and SMG (1:1); SMG, *Salvia macrosiphon* gum.

The rate constant (*k*) and half‐life period (t1/2) parameters are essential for predicting the stability and shelf life of encapsulated powders, and they often exhibit an inverse relationship. These parameters are calculated based on the diagram in Figure 2. In the samples of extract coated with chitosan, CCS, and *S. macrosiphon* gum (SMG), the half‐life period (t1/2) values were determined to be 54.6, 70.7, and 103.4 days, respectively, while the rate constant (*k*) values were 0.0127, 0.0098, and 0.0067, respectively (as shown in Table 3).

Tavakoli et al. (2023) reported in their study that the rate constant (k) and half‐life period (t1/2) for phenolic extract coated with chitosan and *L. perfoliatum* gum, as well as the complex with a 1:1 ratio of these two compounds, were 0.014, 0.0124, 0.0129, and 49.5, 55.9, 53.7 days, respectively. Similarly, Estakhr et al. (2020) reported that the rate constant (*k*) and half‐life period (t1/2) for *F. persica* extract nanoencapsulated with chitosan and locust bean gum ranged from 0.0092 to 0.0145 and from 47.8 to 75.3 days, respectively.

The variations in these results can be attributed to the differences in the type of coating materials and the specific compounds used for nanoencapsulation. These factors play a significant role in determining the stability and longevity of encapsulated powders, and they can be tailored to the desired application and properties of the final product.

### 3.2 Evaluating the effect of the nanoencapsulated extract on the oxidative stability of canola oil

The research findings indicate that the phenolic extract of *P. affinis* coated with CCS (complex with a 1:1 ratio of chitosan and SMG) exhibited the best performance among all the samples in various tests of nanoemulsions. Therefore, this treatment was selected to be added to refined canola oil to assess its impact on oxidative stability. Three concentrations, specifically 100 ppm, 200 ppm, and 300 ppm of this extract, were added to canola oil in both free and nanocoated forms. To facilitate comparison, a concentration of 100 ppm tert‐butyl hydroquinone (TBHQ) was used.

**FIGURE 3:**
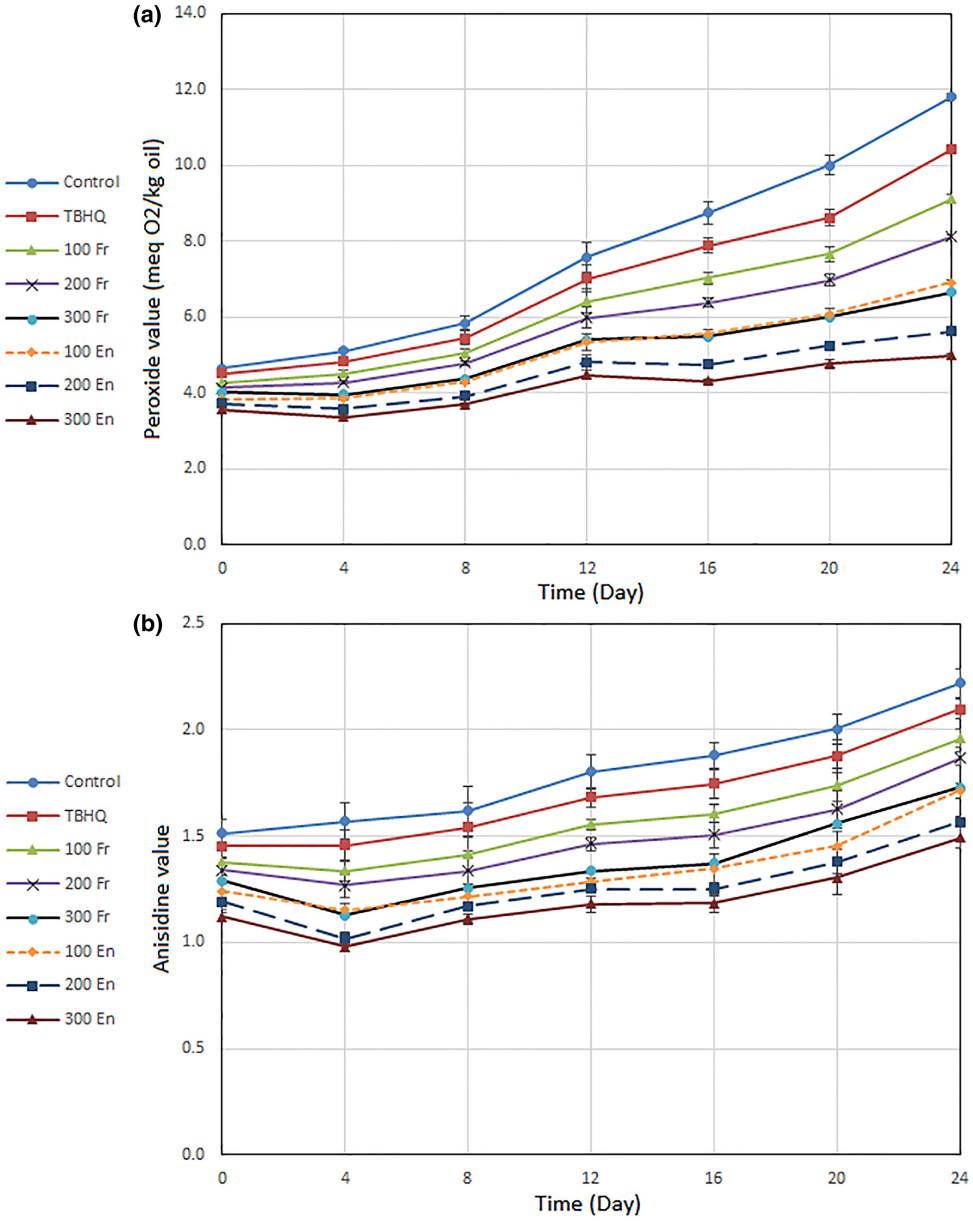
Effect of adding free *affinis* extract (Fr) and nanoencapsulated *P. affinis* extract produced by CCS (En) and TBHQ (100 ppm) on peroxide value (a) and anisidine value (b) of canola oil under accelerated storage at 60°C for 24 days. CCS, complex of chitosan and SMG (1:1).

To evaluate the oxidative stability, changes in the peroxide value and anisidine value were measured, serving as indicators of the initial and secondary oxidation stages. These changes are illustrated in Figure 3. Additionally, the TOTOX value, which represents total oxidation and encompasses both initial and secondary oxidation stages, is presented in Table 4. The TOTOX value was employed to determine the most favorable conditions for the oxidative stability of canola oil.

**TABLE 4:** Effect of adding free *P. affinis* extract and nanoencapsulated *P. affinis* extract produced by CCS and TBHQ (100 ppm) on TOTOX value of canola oil under accelerated storage at 60°C for 24 days.

Time (Day)	Control	Free *P. affinis* extract		
		100 ppm	200 ppm	300 ppm
0	10.8 ± 0.2^a^	10.0 ± 0.1^c^	9.6 ± 0.2^d^	9.3 ± 0.1^e^
4	11.8 ± 0.1^a^	10.4 ± 0.2^c^	9.8 ± 0.1^d^	9.0 ± 0.1^e^
8	13.3 ± 0.3^a^	11.5 ± 0.2^c^	10.9 ± 0.1^d^	10.0 ± 0.1^e^
12	17.0 ± 0.7^a^	14.4 ± 0.6^c^	13.4 ± 0.5^c^	12.2 ± 0.5^d^
16	19.4 ± 0.6^a^	15.7 ± 0.3^c^	14.3 ± 0.3^d^	12.3 ± 0.2^e^
20	21.2 ± 0.5^a^	17.1 ± 0.4^c^	15.6 ± 0.4^d^	13.6 ± 0.3^e^
24	25.8 ± 0.2^a^	20.2 ± 0.2^c^	18.1 ± 0.1^d^	15.0 ± 0.1^e^

Time (Day)	TBHQ	Nanoencapsulated *P. affinis* extract produced by CCS		
		100 ppm	200 ppm	300 ppm
0	10.5 ± 0.1^b^	8.9 ± 0.1^f^	8.6 ± 0.1^g^	8.3 ± 0.1^h^
4	11.1 ± 0.1^b^	8.9 ± 0.1^f^	8.2 ± 0.1^g^	7.7 ± 0.1^h^
8	12.5 ± 0.2^b^	9.8 ± 0.1^e^	9.0 ± 0.1^f^	8.5 ± 0.1^g^
12	15.7 ± 0.7^b^	12.0 ± 0.5^d^	10.9 ± 0.4^e^	10.1 ± 0.2^f^
16	17.5 ± 0.4^b^	12.5 ± 0.2^e^	10.8 ± 0.1^f^	9.8 ± 0.2^g^
20	19.1 ± 0.5^b^	13.6 ± 0.3^e^	11.9 ± 0.3^f^	10.9 ± 0.3^g^
24	22.9 ± 0.2^b^	15.5 ± 0.1^e^	12.8 ± 0.1^f^	11.5 ± 0.1^g^

*Note*: Means ± SD (standard deviation) within a row with the same lowercase letters are not significantly different at *p* < .05.

Abbreviations: CCS, complex of chitosan and SMG (1:1); SMG, *Salvia macrosiphon* gum.

The data presented in Table 4 reveal that the lowest increase in the TOTOX value was observed in canola oil containing 300 ppm of nanoencapsulated extract with CCS (39%). Following this, the oil samples containing 200 ppm of nanoencapsulated extract exhibited a 48% increase in the TOTOX value. Comparatively, canola oil with 300 ppm of free extract had a 61% increase, and canola oil with 100 ppm of nanoencapsulated extract showed a 74% increase. Canola oil with 200 ppm of free extract displayed an 88% increase, while canola oil with 100 ppm of free extract exhibited a 103% increase. Furthermore, canola oil containing 100 ppm TBHQ had a 119% increase, and pure refined canola oil experienced a 139% increase in the TOTOX value.

The results indicate that as the concentration of both free and nanoencapsulated extracts in canola oil increases, its oxidative stability improves. Additionally, the positive impact of the nanoencapsulation process on the performance of the extracts in enhancing the oxidative stability of canola oil has been confirmed. Other studies have also reported the beneficial role of the nanoencapsulation process in enhancing the antioxidant effect of *F. persica* and *M. piperita* extracts in soybean oil (Estakhr et al., 2020; Roshanpour et al., 2021).

In this research, the highest concentration of nanoencapsulated extract (300 ppm) was identified as the most effective treatment for improving the oxidative stability of canola oil. However, it is important to note that increasing the concentration of nanoencapsulated phenolic extract does not always lead to the best conditions for oxidative stability. In a separate study, Tavakoli et al. (2023) investigated concentrations ranging from 100 to 300 ppm of free and nanoencapsulated extracts of *M. aquatic* to enhance the oxidative stability of canola oil. Surprisingly, they found that the concentration of 200 ppm of nanoencapsulated extract yielded the best results, even though the release of phenolic compounds was higher at the 300 ppm concentration of the nanoencapsulated extract. The researchers attributed this phenomenon to the creation of peroxidation conditions resulting from the excessive increase in phenolic compounds at the 300 ppm concentration of the nanoencapsulated extract (Belitz et al., 2009). Furthermore, in this study, both the free and nanoencapsulated extracts of *P.affinis* demonstrated a superior antioxidant effect compared to TBHQ. TBHQ is a potent synthetic antioxidant commonly used, especially in extreme temperature conditions such as frying processes. It is essential to note that the oxidation mechanisms of edible oils differ significantly between low and extreme temperature conditions. In this study, a temperature of 60°C was employed, which led to the observation that the antioxidant effect of *P. affini*s extract was more potent than that of TBHQ. Similar results to those of the present study regarding the antioxidant effect of plant extracts compared to TBHQ have been reported in other studies (Estakhr et al., 2020; Tavakoli et al., 2021). The results of oxidative stability tests clearly demonstrate that the nanoencapsulation process significantly enhances the performance of *P. affinis* extract in canola oil. To better understand the reasons behind these findings, the release rate of total phenolic compounds in extracts coated with CCS during storage at 60°C was measured (Table 5). Upon examination, it became evident that the best conditions were observed in the nanoencapsulated extract with a concentration of 300 ppm, followed by concentrations of 200 and 100 ppm of the nanocoated extract, respectively. The release of a greater quantity of phenolic compounds at a concentration of 300 ppm led to the highest oxidative stability in canola oil in this study. The positive impact of nanoencapsulation on the stability and antioxidant activity of phenolic compounds compared to their free state has been widely reported in various studies (Carneiro et al., 2013). However, it is crucial to note that there is not always a direct relationship between the release of phenolic compounds and oxidative stability. When the level of antioxidants exceeds the optimal threshold, there is a possibility of encountering peroxidative effects rather than antioxidant benefits (Tavakoli et al., 2021, 2023).

**TABLE 5:** The release rate of phenolic compounds (mg/kg) in oil samples from encapsulated powders produced by CCS at levels of 100, 200, and300 ppm.

Time (day)	100 ppm	200 ppm	300 ppm
4	19.3 ± 1.2b	20.1 ± 1.6b	23.0 ± 0.8a
8	40.5 ± 1b	42.0 ± 1.4b	46.3 ± 0.9a
12	46.8 ± 1.4b	48.1 ± 1.6b	56.1 ± 2.7a
16	52.0 ± 1.9b	59.5 ± 2.1a	57.8 ± 1.9a
20	61.5 ± 1.1b	64.9 ± 1.6a	67.8 ± 1.6a
24	73.0 ± 1.7c	78.5 ± 1.1b	80.8 ± 0.7a

*Note*: Means ± SD (standard deviation) within a column with the same lowercase letters are not significantly different at *p* < .05.

Abbreviations: CCS, complex of chitosan and SMG (1:1); SMG, *Salvia macrosiphon* gum.

## 4 CONCLUSION

In this study, the phenolic extract of *P. affinis* underwent a nanoencapsulation process, with chitosan, *S. macrosiphon* gum (SMG), and a chitosan–SMG complex (CCS) used as coatings. The evaluation of nanoemulsions revealed that the CCS‐coated extract performed the best among all samples. Subsequently, this treatment was employed in concentrations ranging from 100 to 300 ppm for enhancing the oxidative stability of canola oil. The results based on the TOTOX value clearly demonstrated the superiority of the encapsulated extract at a concentration of 300 ppm. This enhancement was attributed to the increased release of phenolic compounds from the CCS coating into the canola oil. Overall, the nanoencapsulation process significantly improved the performance of *P. affinis* extract in enhancing the oxidative stability of canola oil.

## AUTHOR CONTRIBUTIONS

**Habib Abbasi:** Funding acquisition (equal); investigation (equal); methodology (equal); software (equal); supervision (equal); validation (equal). **Javad Tavakoli:** Formal analysis (equal); investigation (equal); methodology (equal); project administration (equal); software (equal); supervision (equal); validation (equal); writing – original draft (equal). **Fahimeh Zare:** Conceptualization (equal); data curation (equal); investigation (equal). **Mohsen Salmanpour:** Conceptualization (equal); investigation (equal).

## CONFLICT OF INTEREST STATEMENT

The authors declare no competing interests.

## ETHICS APPROVAL

Not applicable.

## CONSENT TO PARTICIPATE

The authors declare their consent to participate in this article.

## CONSENT TO PUBLISH

The authors declare their consent to publish this article.

## Data Availability

The data that support the findings of this study are available from the corresponding author upon reasonable request.
